# Supplementary figures and images for: Development and validation of a bedside-available machine learning model to predict discrepancies between SaO₂ and SpO₂: Exploring factors related to the discrepancies
Source: PLoS One. 2025 Oct 21;20(10):e0334350. doi: 10.1371/journal.pone.0334350 (PMC12539712; doi:10.1371/journal.pone.0334350)

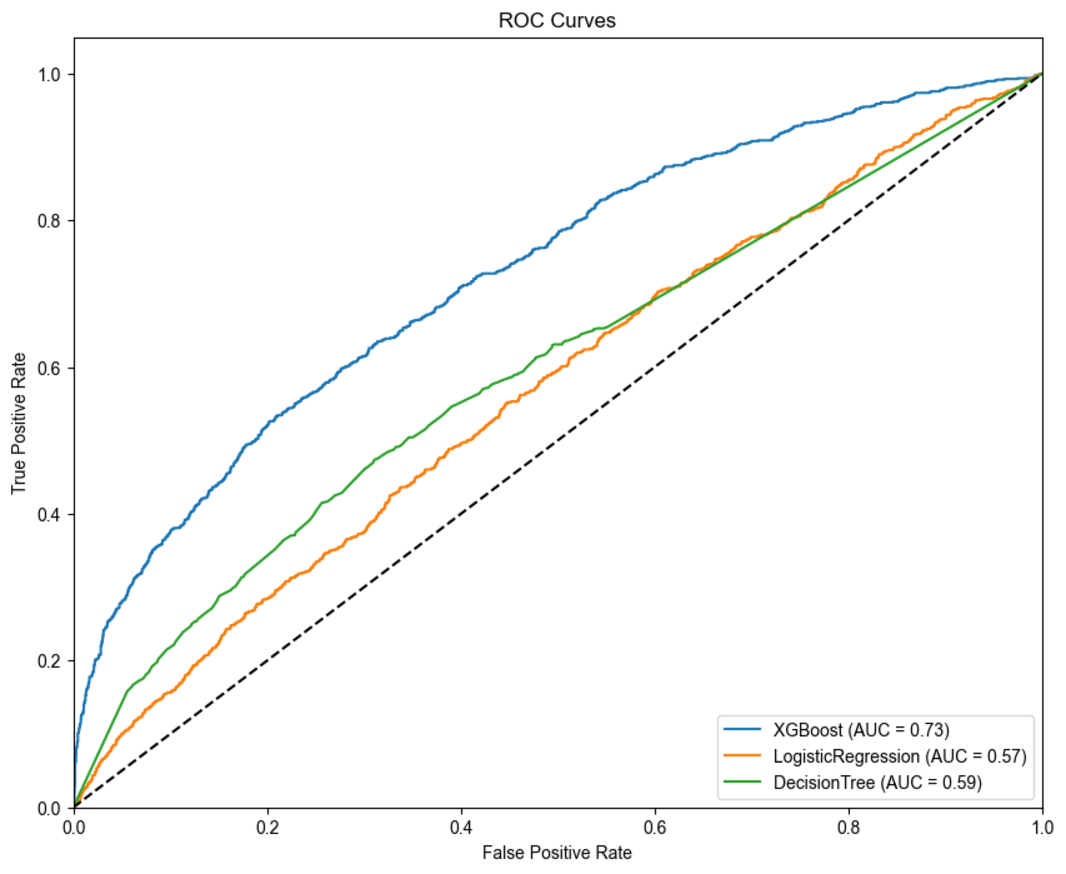

Supplement: S1 Fig — This figure shows the ROC curves and AUC values for three machine learning models: (1) Decision Tree, (2) Logistic Regression, and (3) XGBoost. The ROC curves illustrate each model’s performance by plotting the true positive rate against the false positive rate. The AUC values, displayed on each curve, indicate the overall performance of the models, with higher values representing better discriminatory ability. ROC = Receiver operating characteristic, AUC = Area under the curve, XGBoost = eXtreme Gradient Boosting. (TIF) [file pone.0334350.s001.tif]

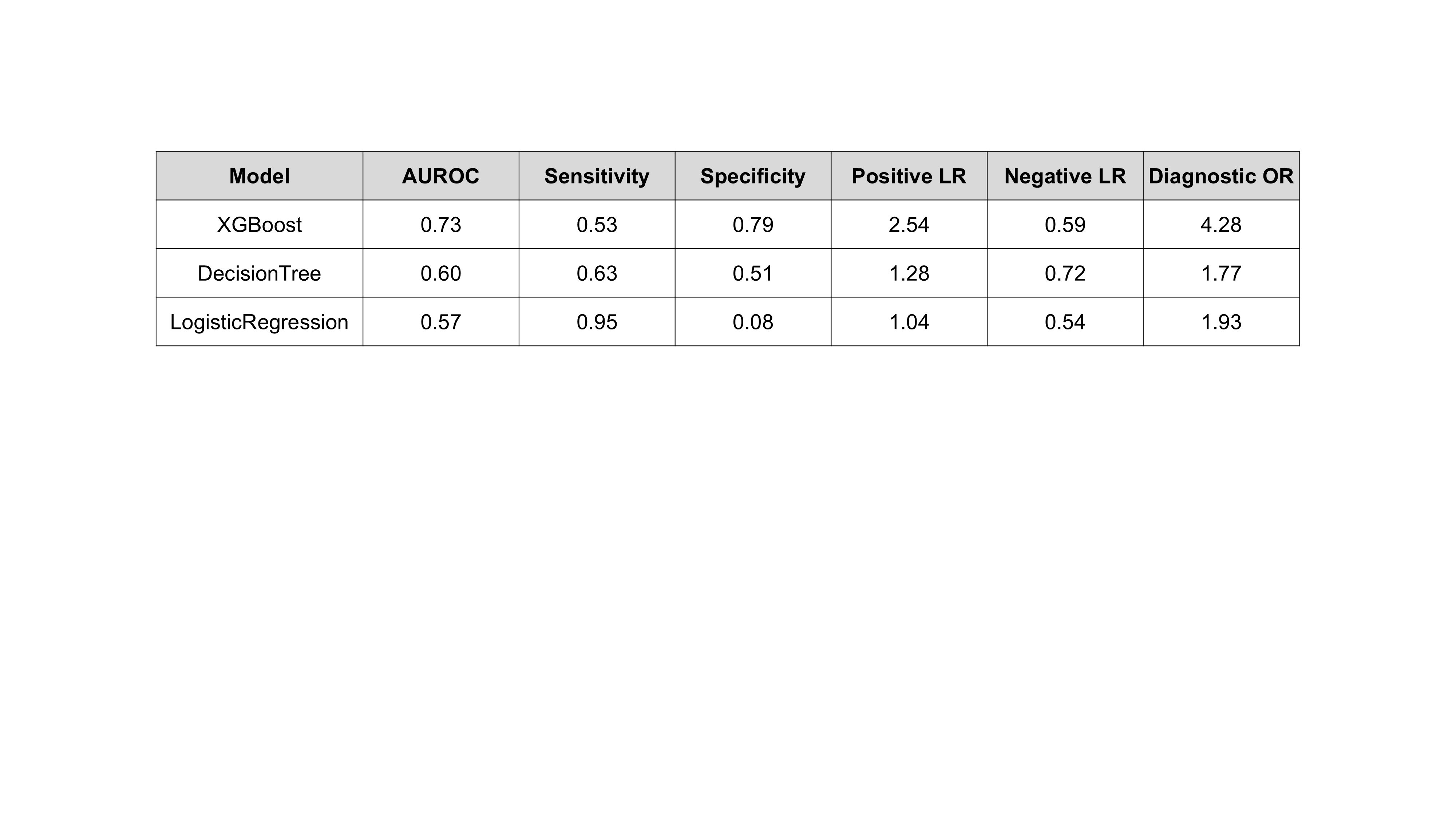

Supplement: S1 Table — AUROC = Area under the receiver operating characteristic curve, XGBoost = eXtreme Gradient Boosting, Positive LR = Positive Likelihood Ratio, Negative LR = Negative Likelihood Ratio, Diagnostic OR = Diagnostic Odds Ratio. (TIF) [file pone.0334350.s002.TIF]

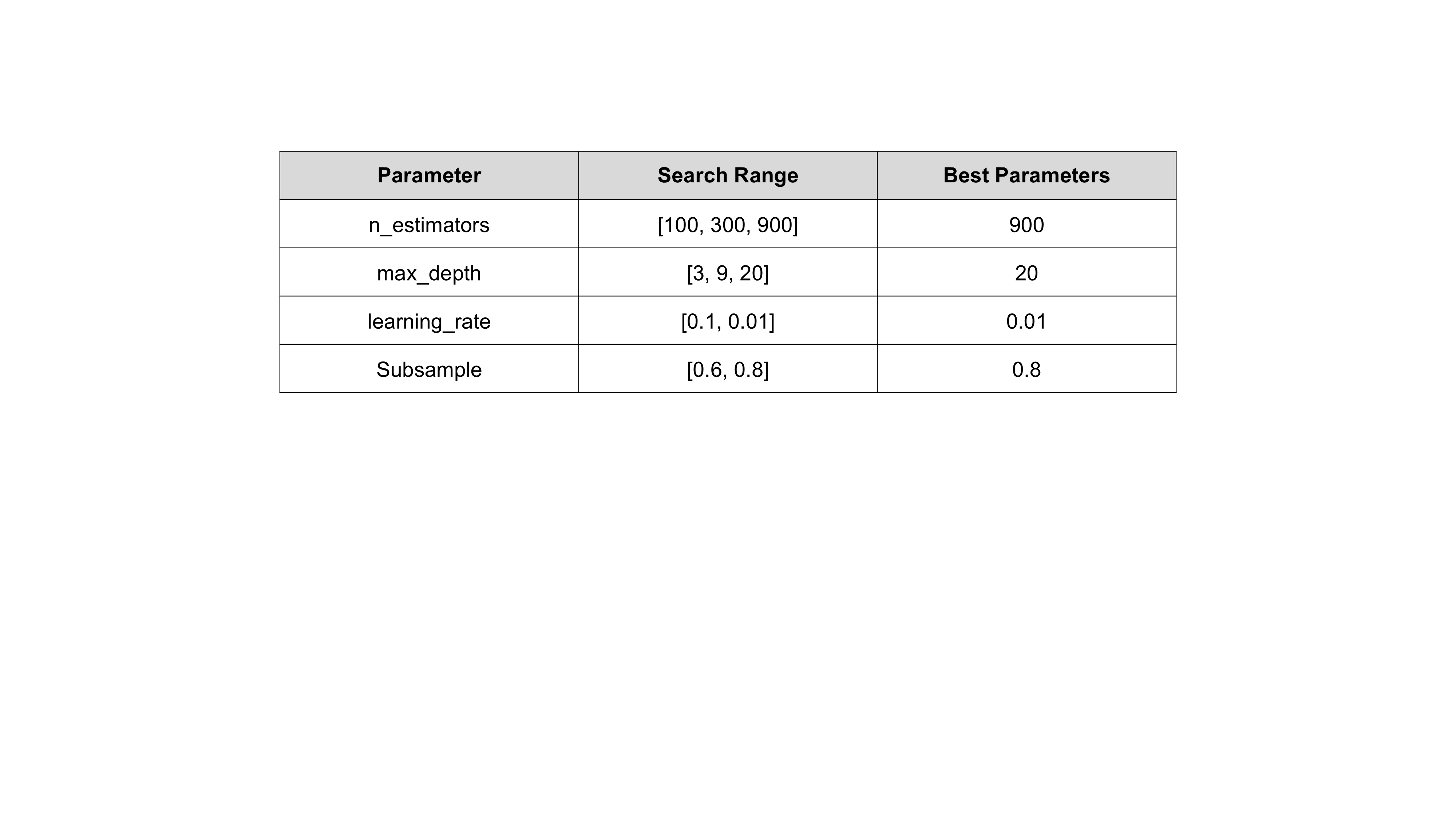

Supplement: S2 Table — XGBoost = eXtreme Gradient Boosting. (TIF) [file pone.0334350.s003.TIF]

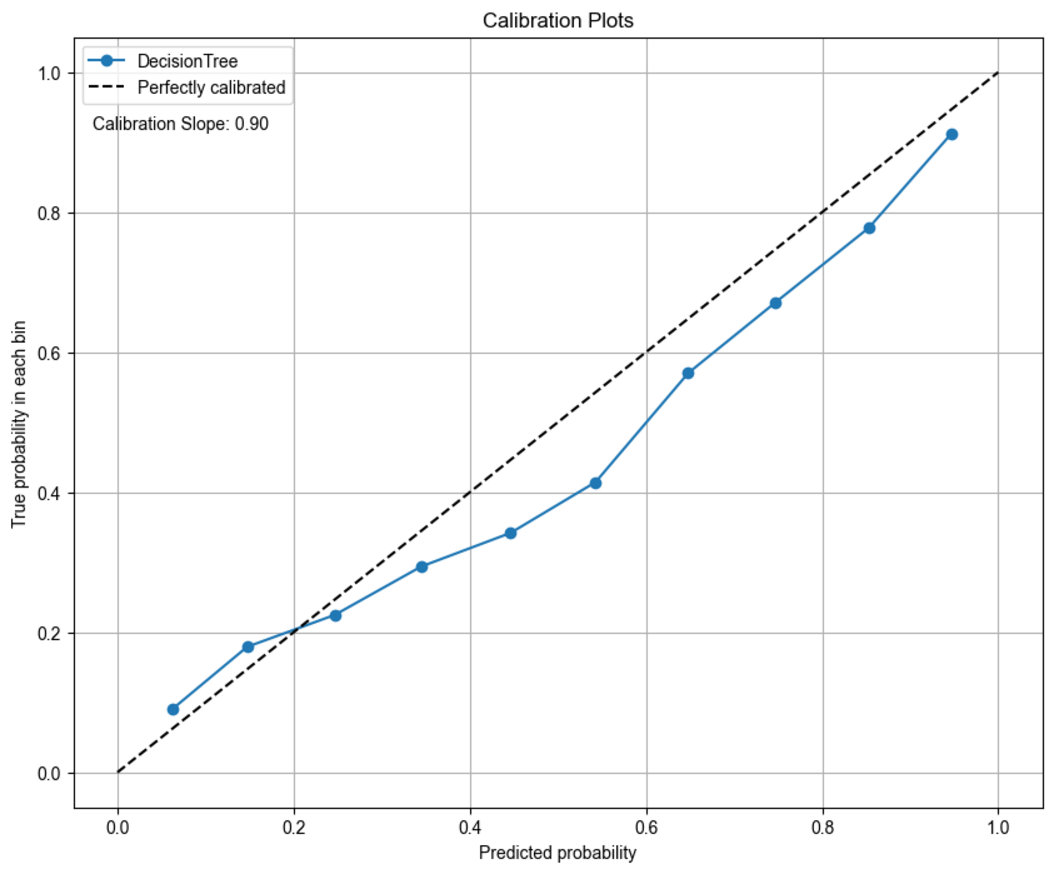

Supplement: S2 Fig — This figure presents the calibration plot and calibration slope for the development cohort using the eICU data set. The calibration plot compares the predicted probabilities of the model with the actual outcomes, illustrating how well the model’s predictions match the observed results. The ideal line represents perfect calibration, where predicted probabilities exactly match the observed frequencies. The calibration slope indicates the agreement between predicted probabilities and actual outcomes. A slope of 1 suggests perfect calibration, while deviations from 1 indicate under- or over-estimation of the predicted risks. eICU = eICU Collaborative Research Database. (TIF) [file pone.0334350.s004.tif]

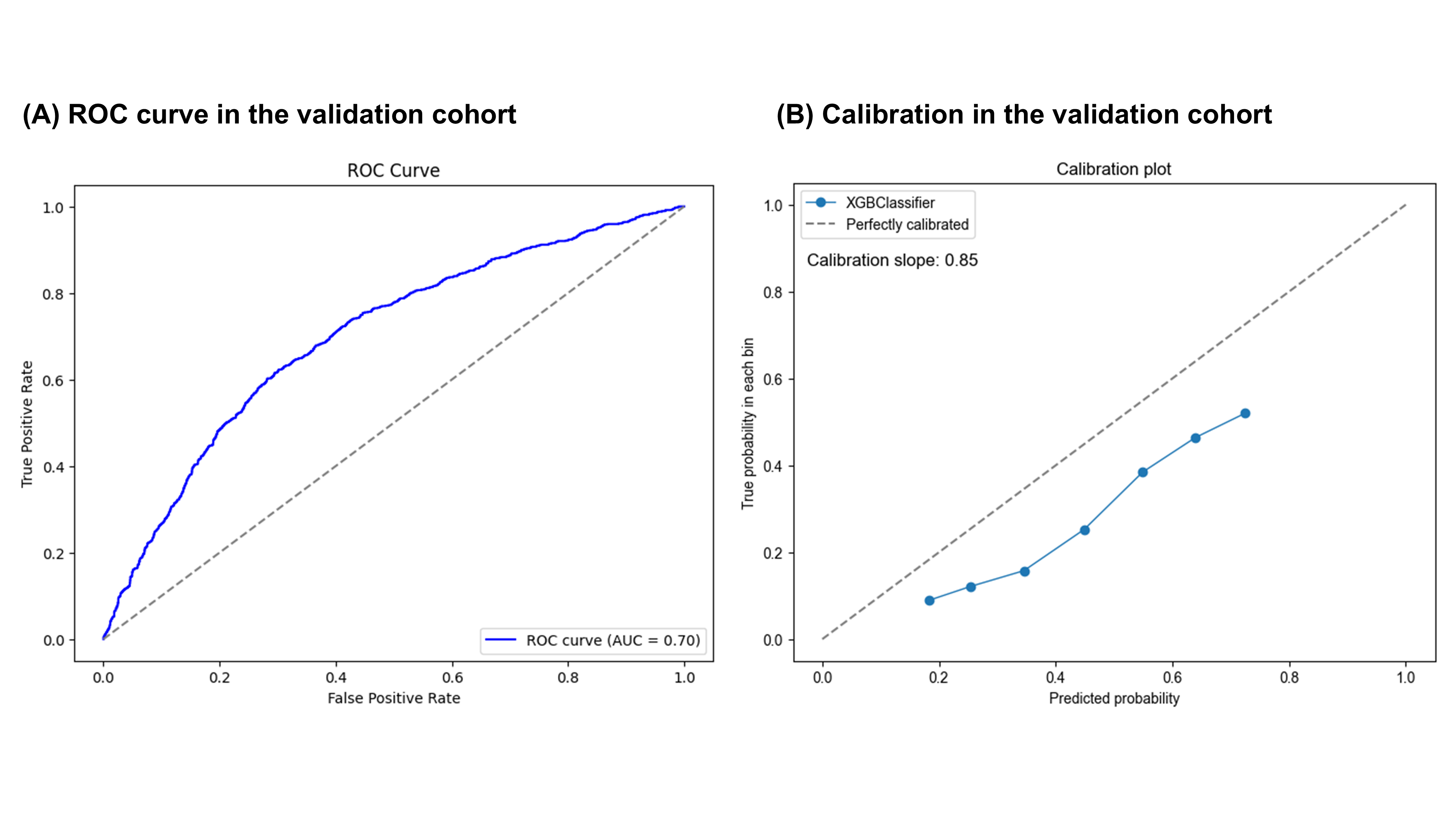

Supplement: S3 Fig — This figure presents the ROC curve and calibration plot for the validation cohort using the MIMIC-IV dataset (2014–2019). The model was trained using data from the eICU database and validated using the MIMIC-IV dataset (2014–2019). ROC = Receiver Operating Characteristic, MIMIC-IV = Medical Information Mart for Intensive Care IV, eICU = eICU Collaborative Research Database. (TIF) [file pone.0334350.s005.TIF]
