# Supplementary material for: Development and validation of a bedside-available machine learning model to predict discrepancies between SaO₂ and SpO₂: Exploring factors related to the discrepancies
Source: PLoS One. 2025 Oct 21;20(10):e0334350. doi: 10.1371/journal.pone.0334350 (PMC12539712; doi:10.1371/journal.pone.0334350)
Supplement: Attachment — Submitted filename: Response to Reviewers (1).docx [file pone.0334350.s007.docx]

Editorial Office

*PLOS ONE*

Dear Dr. Mohsen Mehrabi and Reviewers,

We sincerely appreciate the time and effort that you and the reviewers have dedicated to evaluating our manuscript, "Development and Validation of a Bedside-Available Machine Learning Model to Predict Discrepancies between SaO₂ and SpO₂: Exploring Factors Related to the Discrepancies." We are grateful for the constructive comments and suggestions, which have helped us improve our manuscript. Below, we provide a detailed point-by-point response to the reviewers' comments, along with the corresponding revisions made to the manuscript.

**Reviewer 1**

**Comment 1:**

**The manuscript states that the training phase was conducted using 80% of the dataset. However, it remains unclear how the remaining 20% sample was utilized. A precise description of its role in validation or testing would be beneficial.**

Response:

Thank you for your comment. The dataset was randomly split into 80% for training and 20% for testing. During the training phase, 10-fold cross-validation was conducted exclusively within the 80% training set to optimize hyperparameters and assess model performance. The final model was then evaluated using the remaining 20% as an independent test set. We have clarified this in the manuscript.

Revision:

The **Statistical analysis section** **(page 8, para 1)** has been updated to specify that 80% of the dataset was used for training with cross-validation, while the remaining 20% was used as an independent test set.

*“In the training set (80% random sample), we reduced the risk of algorithmic bias by applying and comparing three machine learning methods, … , Final model evaluation was conducted on the remaining 20% test set, using multiple diagnostic metrics including AUROC, sensitivity, specificity, positive likelihood ratio (Positive LR), negative likelihood ratio (Negative LR), and Diagnostic Odds Ratio”*

**Comment 2:**

**The reported AUC values require further clarification. Specifically, it should be explicitly stated whether they represent the mean AUC obtained during cross-validation or the AUC computed on the 20% test sample.**

Response:

During model training, 10-fold cross-validation was performed within the 80% training set, and the mean AUC from cross-validation was used for model selection. The final model was then evaluated on the independent 20% test set, and the AUC on this test set was separately reported. We have clarified this distinction in the manuscript.

Revision:

Clarifications regarding test set AUC have been added to the **Results section**, **Comparison of three training models subsection** **(page 11, para 2)** as follows: “*Among the three models compared, the XGBoost model demonstrated the highest predictive performance on the test set (20% of dataset) with AUC values of 0.73*”

**Comment 3:**

**A detailed explanation of the hyperparameter tuning process, particularly for the XGBoost model, is warranted. The absence of Table S1 in the provided version of the manuscript prevents verification of whether these details are available.**

Response:

Thank you for your suggestion. To enhance clarity, we have added a new supplementary table (Table S2) that provides a detailed overview of the hyperparameter tuning process for XGBoost, including the search ranges and final values selected. The manuscript now explicitly refers to this table.

Revision:

**The Statistical Analysis section** **(page 8, para 1)** has been updated to include a description of the hyperparameter tuning process for XGBoost. Additionally, a new supplementary table (Table S2) has been added to present the specific parameters tested and their search ranges. This information is now explicitly referenced in the manuscript.

*“Hyperparameter tuning for each model was performed using a grid search combined with 10-fold cross-validation. The specific parameter ranges and selected values for XGBoost are provided in Table S2.”*

**Reviewer 2:**

**Comment 1:**

**Generalizability and Model Performance: The model performs well on the training data (AUROC: 0.73) but poorly on the validation set (AUROC: 0.56). This indicates potential overfitting or dataset bias, which must be addressed.**

Response:

Thank you for your thorough review. As you pointed out, the pre-specified model demonstrated limited transportability on the full external cohort. To investigate whether performance could be improved, we conducted an exploratory hyper-parameter optimization using admissions from 2008–2013 and then evaluated the updated model on the held-out 2014–2019 data. We present this procedure as model updating plus temporal validation and, for transparency, continue to report the original AUROC. The updated model achieved an AUROC of 0.70 (95 % CI 0.68–0.73) with a calibration slope of 0.85. Because the predictor set is restricted to routinely collected variables, the model can be updated in most hospitals with minimal effort. Nevertheless, these findings remain exploratory; we have added a statement noting that additional validation in independent centers and geographical regions is planned and still required.

Revision:

The **Methods section** **(page 8, para 1)** *“As for exploratory analysis to investigate whether performance could be improved in the new independent dataset, we further conducted an exploratory hyper-parameter optimization using admissions from 2008–2013 and then evaluated the updated model on the held-out 2014–2019 data.”*

The **Discussion section** **(page 15, para 2):** *“Compared with a previously reported model that achieved an AUROC of about 0.83 for estimating PaO₂ from SpO₂ in ventilated patients [10], our model shows lower discrimination. Its exclusive reliance on non-invasive inputs may partly explain the reduced precision in estimating SaO₂. Even so, an exploratory temporal-validation analysis produced an AUROC of 0.70, suggesting reasonably stable performance in unseen patients and offering a clinically relevant gauge of generalizability. Further gains may be possible by expanding the training dataset and fine-tuning the timing of the paired measurements.”*

**Comment 2:**

**Feature Importance Analysis: While SHAP values are mentioned, there is limited discussion on how these insights could be used to improve the model.**

Response:

Thank you for your valuable comment. We have expanded the discussion on SHAP values, emphasizing the consistency of top-ranking features across both the training (eICU) and validation (MIMIC) datasets. This consistency strengthens the generalizability of the model. Additionally, we have elaborated on how these insights, supported by similar trends in PDPs, contribute to both model performance and clinical decision-making.

Revision:

In the **Discussion section (page 16, para 1)**, *“SHAP analysis showed that the top-ranked predictors were consistent in the training (eICU) and validation (MIMIC) datasets, indicating that these variables retain their importance across settings and may support wider generalizability. Partial-dependence plots for heart rate, mean arterial pressure, and respiratory rate displayed similar risk gradients in both cohorts, illustrating how changes in vital signs affect the predicted risk and reducing the model’s black-box perception for clinicians. The agreement between SHAP values and PDP trends suggests that these routinely recorded clinical features are key drivers of the predictions.”*

**Comment 3:**

**Clinical Utility: The paper should clarify how the model can be integrated into clinical workflows. Would it be used as a decision-support tool? How should clinicians interpret the results?**

Response:

Thank you for your comment. We have clarified in the discussion that the model can be used as a decision-support tool in ICU settings to help prioritize patients at risk for hypoxemia. The model’s output should guide clinicians in identifying patients who may need closer monitoring.

Revision:

We added to the **Discussion section** **(page 17, para2)** *“Our model demonstrated stable predictive performance in a temporally split validation using the MIMIC-IV dataset, suggesting its potential for integration into clinical workflows as a decision-support tool. In particular, it may help identify patients at risk of hypoxemia, especially those with low perfusion or other critical vital signs. The model outputs can be used to guide the prioritization of patient monitoring in ICU settings, while still relying on clinical judgment and confirmatory tests such as blood gas analysis. Clinicians can use the model’s predictions to support early recognition and intervention for patients who may require closer observation*.

”

**Comment 4:**

**Web-Based Implementation Details: The web-based tool mentioned is not sufficiently described. A brief description or a link to a demonstration would be beneficial.**

Response:

Thank you for your valuable feedback. We have now added a brief description of the web-based tool and its functionality, clarifying how clinicians can input key predictors to obtain a probability estimate for SpO₂–SaO₂ discrepancy.

Revision:

The **Model implementation section** **(page 17, para 3)** *“We have released a lightweight, publicly accessible web application (https://spo2-to-sao2.streamlit.app/) that implements our prediction model. Users manually enter readily available information—vital signs and basic patient characteristics—and the app instantly returns the estimated probability of an SpO₂–SaO₂ discrepancy (≥ 3 percentage points). Although the tool is not yet embedded in hospital information systems and therefore requires hand entry, it still allows clinicians to gauge risk at the bedside and may serve as an engaging proof-of-concept for future, fully integrated deployments.”*

**Comment 5:**

**Statistical Analysis: More robust statistical validation, such as confidence intervals for AUROC, would strengthen the claims.**

Response:

Thank you for your recommendation. We have now included confidence intervals for AUROC values in both the internal and external validation results.

We believe that these revisions have strengthened our manuscript and addressed the concerns raised by the reviewers. We sincerely appreciate the reviewers’ thoughtful comments and the opportunity to improve our work. Please let us know if any further clarifications are required.

Thank you for your time and consideration.

Best regards,

Raito Sato

School of Medical Sciences, University of Fukui
