# Supplementary material for: Development and validation of a bedside-available machine learning model to predict discrepancies between SaO₂ and SpO₂: Exploring factors related to the discrepancies
Source: PLoS One. 2025 Oct 21;20(10):e0334350. doi: 10.1371/journal.pone.0334350 (PMC12539712; doi:10.1371/journal.pone.0334350)
Supplement: Attachment — Submitted filename: RS_Datathon_2rd_Response to Reviewers.docx [file pone.0334350.s008.docx]

Editorial Office

*PLOS ONE*

Dear Dr. Emily Chenette and Reviewers,

We are truly grateful for the time and effort you and the reviewers have invested in evaluating our manuscript, *"Development and Validation of a Bedside-Available Machine Learning Model to Predict Discrepancies between SaO₂ and SpO₂: Exploring Factors Related to the Discrepancies."* We sincerely appreciate your constructive comments and suggestions, which have been invaluable in improving the quality of our work. Please find below our point-by-point responses and the corresponding revisions made to the manuscript.

**Reviewer 2**

**Comment 1:**

**The manuscript uses first-person pronouns (e.g., “we,” “I”) in several places. The authors should revise the writing style to maintain a formal and objective tone.**

Response:

Thank you very much for your valuable comment. In response, we have thoroughly revised the manuscript to eliminate first-person pronouns and ensure a more formal and objective tone throughout the text.

Revision:

No.1: (page 3, para 1)

Original:

*“We aimed to explore the factors related to the discrepancy […]”*

Revised:

*“The aim of this study was to explore the factors related to the discrepancy […]”*

No.2: (page 3, para 2)

Original:

*“we used non-invasive, readily available bedside information (patient demographics, vital signs, vasopressor use, ventilator use) for developing prediction models with three machine learning methods (decision tree, logistic regression, XGBoost). To make the model accessible, we implemented the model as a web-based application. We further explored the contribution of each variable by using partial dependence plots and SHAP value.”*

Revised:

*“non-invasive, readily available bedside information (patient demographics, vital signs, vasopressor use, ventilator use) was used to develop prediction models with three machine learning methods (decision tree, logistic regression, XGBoost). To make the model accessible, the model was deployed as a web-based application. Additionally, the contribution of each variable was explored using partial dependence plots and SHAP value.”*

No.3: (page 3, para 3)

Original:

*“we used 19,804 paired SpO₂ and SaO₂ measurements.”*

Revised:

*“a total of 19,804 paired SpO₂ and SaO₂ measurements were used.”*

No.4: (page 4, para 2)

Original:

*“we developed a machine learning model to predict SpO₂–SaO₂ discrepancy and identified vital signs as key contributors. Our findings underscore […]”*

Revised:

*“a machine learning model was developed to predict SpO₂–SaO₂ discrepancy and identified vital signs as key contributors. These findings underscore […]”*

No.5: (page 5, para 2)

Original:

*“We aimed to develop and validate […] We further investigated the contribution of each variable to the discrepancy.”*

Revised:

*“This study aimed to develop and validate […] The contribution of each variable to the discrepancy was further investigated.”*

No.6: (page 6, para 1)

Original:

*“We employed the eICU database for model development, […]”*

Revised:

*“The eICU database was used for model development, […]”*

No.7: (page 6, para 2)

Original:

*“We included adult patients (aged ≥18 years) in our study, […] The difference between SpO₂ and SaO₂ in our study was required to be no more than 20%. We established the following criteria for inclusion: […] Any records with missing data were excluded from our analysis.”*

Revised:

*“This study included adult patients (aged ≥18 years), […] The difference between SpO₂ and SaO₂ in this study was required to be no more than 20%. The following criteria were established for inclusion: […] Any records with missing data were excluded from this analysis.”*

No.8: (page 8, para 1)

Original:

*“In the training set (80% random sample), we reduced the risk of algorithmic bias by applying and […] We then selected the model with the highest overall diagnostic value for further fine-tuning and validation, […] we further conducted an exploratory hyper-parameter optimization […] In our study, we employed two distinct techniques for model interpretation: […] Our analyses, visualizations, […]”*

Revised:

*“In the training set (80% random sample), the risk of algorithmic bias was reduced by applying […] The model with the highest overall diagnostic value was selected for further fine-tuning and validation […] this study conducted an exploratory hyper-parameter optimization […] In this study, two distinct techniques for model interpretation were employed: […] These analyses, visualizations, […]”*

No.9: (page 9, para 1)

Original:

*“We excluded data points for the following reasons: […] Finally, we identified 4,781 admissions and 19,804 data points suitable for analysis.”*

Revised:

*“Data points were excluded for the following reasons: […] Finally, 4,781 admissions and 19,804 data points were identified as suitable for analysis.”*

No.10: (page 11, para 2)

Original:

*“(A) From the eICU dataset (2014–2015), we identified 70,304 data points that included both SpO₂ and SaO₂ measurements. […] (B) From the MIMIC-IV dataset (2008–2019), we identified 81,797 data points that included both SpO₂ and SaO₂ measurements.”*

Revised:

*“(A) In the eICU dataset (2014–2015), 70,304 data points included both SpO₂ and SaO₂ measurements. […] (B) In the MIMIC-IV dataset (2008–2019), 81,797 data points included both SpO₂ and SaO₂ measurements.”*

No.11: (page 12, para 2)

Original:

*“After comparing other evaluation scores (S1 Table), we selected the XGBoost model for prediction. Additionally, we constructed a calibration plot to further assess the model’s performance.”*

Revised:

*“After comparing other evaluation scores (S1 Table), the XGBoost model was selected for prediction. Additionally, a calibration plot was constructed to further assess the model’s performance.”*

No.12: (page 13, para 2)

Original:

*“We first validated the original XGBoost mode on the MIMIC-IV dataset, […]”*

Revised:

*“The original XGBoost model was first validated on the MIMIC-IV dataset, […]”*

No.13: (page 15, para 2)

Original:

*“In this study, we developed a machine learning model using 19,804 data points […]”*

Revised:

*“In this study, a machine learning model was developed using 19,804 data points […]”*

No.14: (page 15 para 3)

Original:

*“our model shows lower discrimination.”*

Revised:

*“this model shows lower discrimination.”*

No.15: (page 16, para 2)

Original:

*“Our findings about the correlation between the worsening vital signs […]”*

Revised:

*“These findings about the correlation between the worsening vital signs […]”*

No.16: (page 16, para 3)

Original:

*“A key strength of our study lies in offering clear insights […]”*

Revised:

*“A key strength of this study lies in offering clear insight […]”*

No.17: (page 17, para 2)

Original:

*“Our model demonstrated stable predictive performance […]”*

Revised:

*“This model demonstrated stable predictive performance […]”*

No.18: (page 17, para 3)

Original:

*“We have released a lightweight, publicly accessible web application (https://spo2-to-sao2.streamlit.app/) that implements our prediction model.”*

Revised:

*“A lightweight, publicly accessible web application (https://spo2-to-sao2.streamlit.app/) implementing this prediction model has been released.”*

No.19: (page 18, para 1)

Original:

*“Our study has several limitations.* *[…] we restricted our data to vital signs and ABG tests that were recorded within 10 minutes. Second, the database we used for training in this study is solely from the United States, which may limit the generalizability of our results. However, to address this concern and enhance the robustness of our findings, we conducted external validation. Moreover, another limitation of our study is the potential bias arising from using multiple data points from the same patients. This aspect is critical in interpreting our data […]”*

Revised:

*“This study has several limitations. […] the data were restricted to vital signs and ABG tests that were recorded within 10 minutes. Second, the database used for training in this study is solely from the United States, which may limit the generalizability of these results. However, to address this concern and enhance the robustness of these findings, external validation was conducted. Moreover, another limitation of this study is the potential bias arising from using multiple data points from the same patients. This aspect is critical in interpreting the data […]”*

No.20: (page 18, para 2)

Original:

*“a machine learning model was developed to predict when SpO₂ exceeds SaO₂ by 3% or more, while the prediction ability was suboptimal in a different dataset. We identified vital signs (e.g., temperature and heart rate) as factors associated with these discrepancies. Our findings underscore the awareness for hidden hypoxemia and provide the basis of further study to identify hidden-hypoxia in critically ill patients.”*

Revised:

*“a machine learning model was developed to predict when SpO₂ exceeds SaO₂ by 3% or more, while the prediction ability was suboptimal in a different dataset. Vital signs (e.g., temperature and heart rate) were identified as factors associated with these discrepancies. These findings underscore the need for awareness of hidden hypoxemia and provide a basis of further studies to identify hidden-hypoxia in critically ill patients.**”*

**Reviewer 4**

**Comment:**

**The descriptive table in page 10 is not rigorous enough. Only median values and IQR are not enough information. I suggest to look into recent literatures for more detailed descriptive statistics. If the outcome is binary then a bivariate table is warranted.**

Response:

Thank you very much for your constructive comment. In response, we have revised Table 1 to include standard deviations (SD) for continuous variables, as appropriate. Additionally, we have created a new Table 2, which presents a bivariate analysis of each predictor variable stratified by the binary outcome (SpO₂–SaO₂ discrepancy ≥3%). We believe these revisions improve the rigor and clarity of the descriptive statistics in line with recent literature.

We believe these revisions have enhanced the overall quality and clarity of our manuscript and that they adequately address the concerns raised by the reviewers. We are grateful for the valuable feedback and the opportunity to further improve our work. Please do not hesitate to let us know if any additional information or clarification is required.

Thank you for your time and consideration.

Best regards,

Raito Sato

School of Medical Sciences, University of Fukui
